# Design of an on-Chip Room Temperature Group-IV Quantum Photonic Chem/Bio Interferometric Sensor Based on Parity Detection

**DOI:** 10.3390/nano10101984

**Published:** 2020-10-07

**Authors:** Francesco De Leonardis, Richard A. Soref, Vittorio M. N. Passaro

**Affiliations:** 1 Photonics Research Group, Department of Electrical and Information Engineering, Politecnico di Bari, 70125 Bari, Italy; francesco.deleonardis@poliba.it; 2 Institute for Photonics and Nanotechnologies (CNR-IFN), Department of Physics, via E. Orabona n. 4, 70125 Bari, Italy; 3 Department of Engineering, University of Massachusetts, Boston, MA 02125, USA; soref@rcn.com

**Keywords:** quantum sensing, group IV photonics, integrated optics, nanophotonics, interferometer, parity detection

## Abstract

We propose and analyze three Si-based room-temperature strip-guided “manufacturable” integrated quantum photonic chem/bio sensor chips operating at wavelengths of 1550 nm, 1330 nm, and 640 nm, respectively. We propose design rules that will achieve super-sensitivity (above the classical limit) by means of mixing between states of coherent light and single-mode squeezed-light. The silicon-on-insulator (SOI), silicon-on-sapphire (SOS), and silicon nitride-on-SiO_2_-on Si (SiN) platforms have been investigated. Each chip is comprised of photonic building blocks: a race-track resonator, a pump filter, an integrated Mach-Zehnder interferometric chem/bio sensor, and a photonic circuit to perform parity measurements, where our homodyne measurement circuit avoids the use of single-photon-counting detectors and utilizes instead conventional photodetectors. A combination of super-sensitivity with super-resolution is predicted for all three platforms to be used for chem/bio sensing applications.

## 1. Introduction

Constructing quantum photonic chips using a group-IV approach is becoming recognized as a highly capable way to build a quantum photonic system-on-a-chip, that is, a quantum photonic integrated circuit (QPIC). Classical and quantum group IV PICs are built upon a silicon substrate, but there are actually several useful silicon-based photonic “platforms” available today, notably silicon-on-insulator (SOI), silicon-on-sapphire (SOS), and silicon nitride-on-SiO2-on Si (SiN). In our view, the practicality of a QPIC chip depends primarily upon operating the chip at room temperature. Taking the chip design a step further, and assuming the 300K constraint, then the chip practicality depends upon the choice of the platform, because, in terms of quantum performance metrics, there will be a “hierarchy of performance” among SOI, SOS, SiN, with the best one determined initially by simulations. Each platform has its “sweet spot” of operation, which means that each platform has its optimum wavelength-of-operation where two high-performance integrated components are available: the 300K nonlinear-optics quantum light source and 300K waveguide-integrated photodetectors. Our research and recent literature show that the optimum wavelengths are 1550 nm for SOI, 1330 nm for SOS and 640 nm for SiN, respectively. Taking that result into account, we have performed a theoretical analysis in order to demonstrate the feasibility of the use of these platforms in the area of quantum sensing—in particular, the on-chip sensing of chemical and biological agents. Our primary research goal is to design and simulate QPIC chips that give higher or much higher sensitivity than the classical sensitivity limit. We analyze fully integrated room-temperature chem/bio sensor chips that use (1) the mixing between a squeezed vacuum light state and a coherent light state, and (2) parity detection to provide super-sensitive phase estimation.

The paper is organized into the following sections: advanced quantum sensing techniques, super-sensitivity in phase estimation, chem-bio QPIC chip-sensor architectures, design rules for three platforms, parity-based detection, platform selection, and conclusions.

## 2. Advanced Quantum Sensing Techniques

Considerable research interest has been focused on the use of quantum phenomena within the field of quantum sensing for a broad range of applications. While for static signals the main figure-of-merit is the sensitivity, for time-dependent signals it is the spectral resolution, i.e. the ability to resolve two different frequencies [1]. In this context, new super-resolution methods that rely on quantum features, have been recently developed [1]. However, in the chemical/bio sensing scenario, exceptional sensitivity is required. Indeed, in a review paper [2] the authors outline that the development of ultra-sensitive and ideally label-free detection schemes are crucial for analyzing the aggregation state of proteins, fundamental for neurodegenerative disease. Therefore, the exploitation of quantum systems to estimate unknown parameters leads to surpassing the precision limits that can in principle be obtained using only classical resources. In this sense, the aim of Quantum Metrology is to find schemes and methods that reach the ultimate fundamental bounds on estimation precision. Thus, in the Quantum Metrology context, we obtain phase super-resolution if the phase derivative of the measured output is larger than the case of classical light. Moreover, phase super-sensitivity is reached if the phase uncertainty in the phase measurement is lower than the classical limit induced by the central limit theorem. 

Generally speaking, any measurement consists of three parts: the preparation of a probe, its interaction with the system to be measured, and the probe readout. This process is affected by statistical or systematic errors. In particular, the statistical error source can have an accidental or fundamental nature. The former depends on the imperfections of the probes or measurement system, while the latter derives from the Heisenberg uncertainty relations. Given an unknown parameter to be estimated and m classical probes (with m≫1), the classical limit, called the Standard Quantum Limit (SQL), imposes that the estimation precision scales as $m^{-1/2}$. The SQL is not a fundamental quantum mechanical bound as it can be surpassed by using “non-classical” strategies. In this context, the ultimate limit on estimation precision is called the Heisenberg Limit (HL) and it scales as $m^{-1}$, improving the precision by a factor $m^{1/2}$ with respect to the classical approach. Thus, in the quantum sensing scenario, we realize the super-sensitivity if the quantum system can beat the SQL. However, the use of the quantum approach does not guarantee by itself the super-sensitivity behavior. Indeed, two basic quantities, such as the Fisher Information and the Cramér-Rao bound (CRB), characterize a general estimation process. In this sense, any quantum system must minimize the CRB causing it to be lower than SQL and approaching HL. The basic concepts behind quantum metrology, with particular attention to phase estimation, have been reviewed [3,4], where the authors describe the current state-of-the art in terms of platforms, quantum resources, and current experimental and theoretical challenges. 

Although several physical quantum systems have been developed, it seems that photonic systems represent ideal probes for a large number of metrological tasks. In this context, a large number of quantum systems based on discrete optics have been proposed in literature (as discussed in the following section) in order to induce super-sensitivity in the phase estimation. However, despite the large number of research works, the open issue is the implementation of quantum integrated photonic sensors with quantum-enhanced performances in the presence of noise. In this scenario, recent advances towards on-chip chem/bio quantum photonic sensing platforms have been discussed based on the N00N state [2], where a potentially promising sensor testbed to achieve super-sensitivity is presented. Those authors present a sensor design comprising two fiber-coupled GaAs chips, a light source chip, and a chem/bio chip. Entangled photons are generated by the excitation of quantum dots, embedded within the chip waveguide at cryogenic temperature (4 K), in order to prevent the phonon-induced broadening. The generated photons are coupled via fiber optics to the 300K sensor chip containing an integrated Mach-Zehnder interferometer (MZI) where a sensing window is open on one MZI arm. Although the light source chip proposed [2] is cryogenic, it overcomes the disadvantage of employing squeezed light, typically based on nonlinear schemes such as Spontaneous Parametric Down Conversion (SPDC) and Spontaneous Four Wave Mixing (SFWM). These schemes are probabilistic, and thus require relatively high pump power that needs to be filtered out prior to sending the generated photons to the sensing area. Moreover, the authors outline that the presence of such off-chip filtering represents a barrier to chip integration. However, the device imperfections, loss, and instability can reduce the interference visibility of the N00N state (discussed in the next section), thereby compromising the super-sensitivity behavior. In this sense, we guess that a mixing performed between squeezed and coherent states of the light can represent an efficient way to guarantee super-sensitivity at room temperature. This approach is investigated in the present paper. In addition, the parity detection scheme adopted here adds robustness to the integrated sensor chip. Moreover, we think that the issue of the off-chip pump filtering, outlined in [2], can be solved by adopting the integrated filter solution proposed in [5], where −90 dB of pump transmission has been proven. 

## 3. Super Sensitivity in Phase Estimation

Quantum interference of light plays a crucial role in high-precision quantum sensing [6], optical quantum computation [7], and quantum state tomography [8]. A typical measurement setup in a quantum-interferometric application is a balanced Mach-Zehnder interferometer containing two beam splitters (BS), which is used with phase sensing. When a phase shift (φ) is induced on one arm of the MZI, the output records an oscillatory fringe pattern with a periodicity given by half wavelength (λ/2), usually referred as the “Rayleigh criterion” for phase measurements. However, this limit can be surpassed using different types of quantum states or measurement schemes [1], [9,10,11]. In this sense, the goal is to recover the phase value by measuring the signals emerging from the MZI, while using a limited amount of resources by setting an upper limit on the average (maximum) number N of photons incoming at the MZI inputs. 

Generally speaking, phase super-resolution and phase super-sensitivity are attributed to quantum behavior in interferometry. The arguably best-known quantum approach to observe such fringe narrowing uses N00N states at the MZI inputs $|\psi \rangle =1/\sqrt{2}\left({|N,0\rangle }_{A,B}\pm {|0,N\rangle }_{A,B}\right)$, where A and B represent the mode paths [9], [12,13,14]. Thus, assuming that all photons in the B path acquire a phase shift of φ, the quantum state inside the MZI becomes $|\psi \rangle =1/\sqrt{2}\left({|N,0\rangle }_{A,B}\pm e^{jN\varphi }{|0,N\rangle }_{A,B}\right)$. If this state is sent onto the second beam splitter (MZI output) and N-fold coincidence is measured by means of an array of single-photon counting detectors, the measured probability can be expressed as $p_{\pm }\left(\varphi \right)=0.5f\left(1\pm Vcos\left(N\varphi \right)\right)$, where V is the visibility of the fringe pattern and $f=\eta_{p}\eta_{D}^{N}$, with $\eta_{D}$ the detector efficiency and $\eta_{p}$ the constant of proportionality depending on both the input state and the detection events [14]. In addition, the visibility is defined as $V=\left(I_{max}-I_{min}\right)/\left(I_{max}+I_{min}\right)$, being $I_{min}$ and $I_{max}$ the signal oscillation minimum and maximum, respectively. The probability $p_{\pm }\left(\varphi \right)$ oscillates N times faster than the measured intensity in the classic case, $I\left(\varphi \right)=0.5I_{0}\left(1+cos\left(\varphi \right)\right)$, so showing clearly the super-resolution effect. Moreover, the probability $p_{\pm }\left(\varphi \right)$ does not impose any limitation on the value of N for all applications where the super-resolution is the performance parameter of interest, such as optical lithography [15], matter-wave interferometry [16], and radar ranging [17]. In this context, even higher N00N states (N = 5) have been realized by mixing quantum and classical light and using the discrete optics approach, where visibility values around 95%, 86%, 74%, 42% for N = 2,3,4,5, respectively, have been obtained [18]. However, this faster oscillation itself is not a proof of super-sensitivity, defined as the reduced phase uncertainty compared to the SQL. In the sensing scenario, the signature of quantum super-resolution does not quantify improved performance beyond classical interferometry. Thus, the employment of the quantum approach instead of classical can be justified only if the super-sensitivity is guaranteed. Generally speaking, N00N states can induce the effects of super-resolution and super-sensitivity simultaneously, offering a sensitivity with Heisenberg scaling, 1/N [2]. However, theoretical and experimental investigations have demonstrated that resolution and sensitivity compete to each other, as induced by the Cramer-Rao bound (CRB) [14], which limits the precision of the phase estimation: $\Delta \varphi \geq 1/\sqrt{F\left(\varphi \right)}$, where $F\left(\varphi \right)=\underset{j}{\sum }\left(1/p_{j}\left(\varphi \right)\right){\left|\partial p_{j}/\partial \varphi \right|}^{2}$ is the Fisher information. Thus, to overcome the SQL, the condition ${\left(1/\sqrt{F\left(\varphi \right)}\right)}_{min}\leq 1/\sqrt{N}$ must be satisfied, inducing for the N00N case in the following relationship:

(1) $$V>V_{th}=\sqrt{\frac{1}{fN}}$$

where $V_{th}$ is the threshold visibility.

Thus Equation (1) imposes a constraint on the use of higher-order N00N states, since the visibility decreases while the value of N increases. Moreover, even if we assume the optimum condition for the state 2002, i.e., V = 1, Equation (1) requires that the detector efficiency must satisfy the condition $\eta_{p}\eta_{D}^{2}\geq 0.5$. Thus, the best N00N candidate in order to simultaneously achieve both super-resolution and super-sensitivity is the state 2002, providing the condition $\eta_{p}\eta_{D}^{2}\geq 0.5$. Another limitation of the direct production of N00N states via SPDC or SFWM in Group IV structures is the decreasing probability for the creation of higher states. As a result, a reduction of the parameter $\eta_{p}$ is recorded with a consequent increase in the threshold visibility.

**Figure 1:**
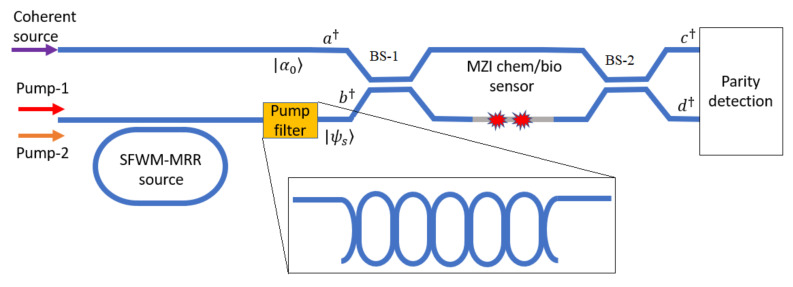
Chem/Bio sensor architecture based on the mixing between squeezed and coherent states. Inset: pump filter.

In order to overcome these limitations, several approaches based on squeezed states of light have been proved to represent a powerful and practical way to achieve the super-sensitivity behavior. In this context, it has been recently suggested to employ the mixing between a vacuum squeezed state and a coherent state of light in combination with Gaussian measurements to achieve super-sensitivity [19]. The experimental setup, realized using discrete optics, recorded a 1.7-fold improvement in the sensitivity with respect to the SQL. However, there has been a growing interest in recent years on the parity of states as a useful observable for various applications such as sensing and remote sensing. In particular, parity has been shown to be a very useful method of detection in interferometry for a wide range of input states. Essentially, parity measurement detects whether the number of photons in a given output mode is even or odd. The parity operator is given by [19,20,21,22,23,24]:

(2) $$\hat{\Pi }={\left(-1\right)}^{c^{†}c}$$

where $c^{†}c$ is the photon number operator (see Figure 1).

As outlined in [23], a potential advantage of the parity detection might be the quantum metrology in the presence of loss. Many of the states and detection schemes used to achieve phase super-sensitivity degrade in lossy environments, limiting the performances. For example, considering the N00N states and the coincidence measurements, all device imperfections and losses can be incorporated into the parameter $\eta_{p}$. Thus, any degradation of $\eta_{p}$ will induce an increase of the threshold visibility, compromising the possibility of obtaining the super-sensitive behavior. Moreover, the detection setup requires a number of detectors proportional to the order of the coincidence probability to be measured. In this sense, the receiver based on the parity detection can be the same, independent of the several different input states which can be selected on the basis of the environmental conditions [23].

In this context, the use of two-mode squeezed vacuum states (produced by means of a crystal with high $\chi^{\left(2\right)}$ nonlinearity) in combination with parity detection to attain simultaneously both super-resolution and super-sensitivity, has been proposed [23,24,25]. Two other common approaches, based on the parity detection, involve the coherent and squeezed vacuum lights [24] and coherently the stimulated parametric down-conversion [26]. On the basis of the analysis reported here, it seems that the approach involving only N00N states can be too critical. Thus, we believe that the mixing between single-mode squeezed and coherent states represents an efficient and robust way to realize an integrated super-sensitive quantum chip.

## 4. Chem/bio QPIC Sensor-Chip Architectures 

The goal of this section is to determine the design rules for the integrated quantum chem/bio sensor based on the mixing between coherent and squeezed vacuum light and operating in the super-sensitivity regime. In this context, we will assume three different technological platforms: SOI, SOS, and SiN. In particular, we assume a strip waveguide structure having height H, and width W. Thus, a silicon waveguide on the silicon dioxide and sapphire layers is considered for SOI and SOS platforms, respectively, while Si-enriched SiN on SiO_2_ is assumed for the SiN platform. The manufacturing process of these platforms is CMOS-compatible, moreover the increased silicon content in SiN platform provides a reduction of the tensile stress and an increase of Kerr refractive index of about 5 times with respect to the stoichiometric silicon nitride [27,28,29].

Figure 1 shows the integrated quantum photonic chip architecture and operation scheme. The chip area consists of three waveguide-connected sections for: (i) the generation of quantum states, (ii) the interferometer chem/bio sensor, and (iii) the detection circuit. Basically, in the generation section, the photonic circuit consists of a bus waveguide into which two external pump laser beams are coupled to create the photon sources. Using those co-traveling lights, the degenerate SFWM process is induced in the side-coupled race-track ring resonator (MRR) in order to generate the single mode squeezing. A filtering stage having an architecture as proposed in [5] is included in order to filter out the residual pump light before sending the signal photons to the sensing area. The sensor chip is realized by means of an interferometer MZI composed of two equal arms where an evanescent-wave waveguide cladding “window” is opened on one of two arms in order to induce the interaction between photons and the analyte molecules on the surface of the waveguide. Thus, a phase shift is induced in the MZI via changes of the effective refractive index due to the analyte. A coherent laser beam from an external laser is end-coupled to a second strip input-waveguide to feed the second input of the MZI. Therefore, the squeezed $|\psi_{s}\rangle$ and coherent $|\alpha_{0}\rangle$ states are injected at the two inputs of the MZI device. Furthermore, most of the photons in this scheme originate from the coherent (classical) light source, which is practically unlimited in intensity, since it is produced by an out-of-chip laser. That eliminates the need to operate with SFWM sources having a high squeezing parameter and high levels of pump power. As discussed below, high pump powers induce detrimental effects such as Two Photon Absorption (TPA), Free Carrier Absorption (FCA), and resonance shift as induced by thermal and plasma effects.

As before mentioned, we adopt the degenerate SFWM process, where degenerate pairs (signal photons) are generated using a dual-wavelength pump scheme $\left(p_{1},p_{2}\right)$. The energy conservation and the phase matching require $2\omega_{s}=\omega_{p1}+\omega_{p2}$ $(\omega_{p2}-\omega_{s}=\omega_{s}-\omega_{p1}=\Delta \omega$) and $\Delta \beta =2\beta_{s}-\beta_{p1}-\beta_{p2}$ = 0, respectively. In the following analysis we assume $P_{1}=P_{2}=P_{0}$, where $P_{1}\;$ and $P_{2}$ represent the powers for the pumps $p_{1}$ and $p_{2}$, respectively. The quantum state at the MZI input is given by:

(3) $$|\psi_{in}\rangle =|\alpha_{0}\rangle ⊗|\psi_{s}\rangle =\sum_{n=0}^{\infty }\sum_{p}^{\infty }C_{p}e^{\frac{-{\left|\alpha \right|}^{2}}{2}}\alpha_{0}^{n}\frac{{\left(a^{†}\right)}^{n}}{n!}\frac{{\left(b^{†}\right)}^{p}}{\sqrt{p!}}$$

where $a^{†}$(a), $b^{†}$(b) are the creation (annihilation) operators for photons in spatial mode A (top waveguide) and B (bottom waveguide), respectively. The parameter $\alpha_{0}=\left|\alpha_{0}\right|e^{j\theta_{c}}$ characterizes the coherent state, where ${\left|\alpha_{0}\right|}^{2}$ represents the average number of photons in the coherent light. The coefficient $C_{p}$ depends on the squeezing parameter (r), according to the following formula:

(4) $$C_{p}=\left\{\begin{array}{ll} {\left(-1\right)}^{p/2}{\left[\frac{p!\tanh^{p}\left(r\right)}{2^{p}{\left[\left(p/2\right)!\right]}^{2}\cosh\left(r\right)}\right]}^{1/2} & p\;\mathrm{even}\\ 0 & p\;\mathrm{odd} \end{array}\right.$$

**Figure 2:**
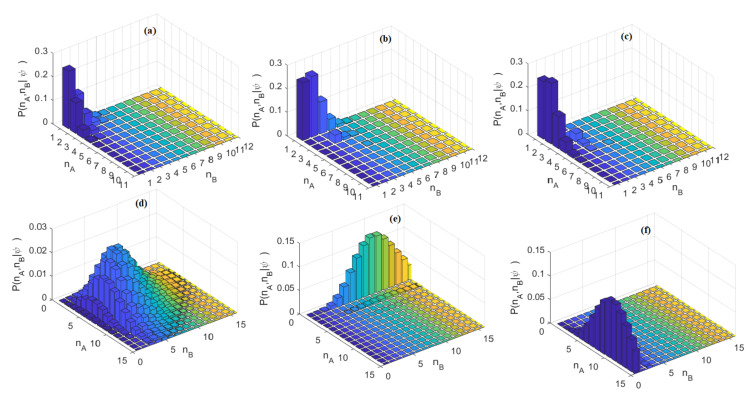
Probability of detecting $n_{A}$ photons in the mode A and $n_{B}$ photons in the mode B; (**a**) After BS-1, r = 0.9, $n_{co}=n_{sq}$; (**b**) MZI output, r = 0.9, $n_{co}=n_{sq}$, φ = 0; (**c**) MZI output, r = 0.9, $n_{co}=n_{sq}$, φ = π; (**d**) After BS-1, r = 0.3, N = 10; (**e**) MZI output, r = 0.3, N=10, φ = 0; (**f**) MZI output, r = 0.3, N=10, φ = π.

Figure 2a–f show the probability of detecting $n_{A}$ photons in the mode A and $n_{B}$ photons in the mode B, after the first beam splitter (BS-1) and at the MZI output, assuming φ = 0 and φ = π, respectively. The probability of detecting $n_{A}$ photons in the mode A and $n_{B}$ photons in the mode B ($P\left(n_{A},n_{B}|\psi \right)$) is calculated by means of the relationship: $P\left(n_{A},n_{B}|\psi \right)={\left|n_{A},\langle n_{B}|\psi \rangle \right|}^{2}$, where ψ is obtained by Equation (3), using the relationship between the operators $a^{†}$, $b^{†}$ and the relative creation operators at the output of the BS-1 or MZI [26]. In the simulations, we have assumed either $n_{co}=n_{sq}$ or $n_{co}>n_{sq}$. The total average photon number is given by $N=n_{co}+n_{sq}={\left|\alpha_{0}\right|}^{2}+sinh^{2}\left(r\right)$.

The Wigner function relevant to the input state is given by [24]:

(5) $$W_{in}\left(\alpha ,\alpha_{0};\beta ,r\right)=\frac{2}{\pi }e^{-2{\left|\alpha -\alpha_{0}\right|}^{2}}\cdot \frac{2}{\pi }e^{\left[-2{\left|\beta \right|}^{2}conh\left(2r\right)-\left(\beta^{2}+\beta^{*2}\right)\sinh\left(2r\right)\right]}$$

where the phase difference between the squeezed and coherent state is taken into account in the phase term $\theta_{c}$. The Wigner function at the MZI output ($W_{out}\left(\alpha_{out},\beta_{out}\right)$) can be obtained by means of the scattering matrix of the MZI device, by applying the following variable transformation:

$$\begin{array}{l} \alpha \to -je^{j\left(\frac{\varphi }{2}\right)}\left[\alpha_{out}\sin\left(\frac{\varphi }{2}\right)+\beta_{out}\cos\left(\frac{\varphi }{2}\right)\right]\\ \beta \to -je^{j\left(\frac{\varphi }{2}\right)}\left[\alpha_{out}\cos\left(\frac{\varphi }{2}\right)-\beta_{out}\sin\left(\frac{\varphi }{2}\right)\right] \end{array}$$

Finally, according to the theory proposed in [24], the expectation value of the parity operator is given by:

(6) $$\langle \hat{\Pi }\rangle =\frac{e^{\left[-n_{co}\left(\frac{\sqrt{n_{sq}^{2}+n_{sq}}\;\sin^{2}\left(\varphi \right)\cos\left(2\theta_{c}\right)-\cos\left(\varphi \right)}{n_{sq}\sin^{2}\left(\varphi \right)+1}+1\right)\right]}}{\sqrt{n_{sq}\;\sin^{2}\left(\varphi \right)+1}}$$

with the following phase sensitivity [24]:

(7) $$\Delta \varphi =\sqrt{\frac{1}{2n_{co}\sqrt{n_{sq}^{2}+n_{sq}}\cos\left(2\theta_{c}\right)+2n_{co}n_{sq}+n_{sq}+n_{co}}}$$

**Figure 3:**
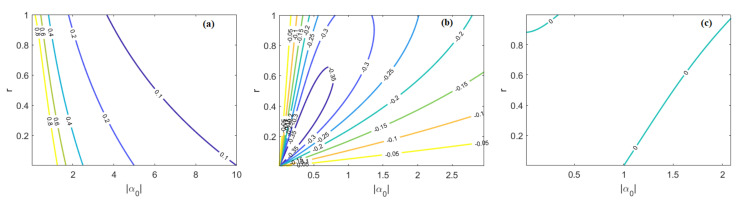
(**a**) Level curves of $\Delta \varphi_{min}$ in the plane ($\left|\alpha_{0}\right|$, r); (**b**) level curves of ($\Delta \varphi_{min}-1/\sqrt{N}$) in Table 1; (**c**) locii of the points where $\Delta \varphi_{min}$ approaches HL.

Figure 3a shows the level curves of the minimum sensitivity ($\Delta \varphi_{min}$; with $\theta_{c}$ = 0) in the plane ($\left|\alpha_{0}\right|$, r). The plot records that, for given values of the squeezed parameter (r), the minimum sensitivity decreases when the average photon number in the coherent state is increased. The level curves for the difference between the minimum sensitivity and the standard quantum limit ($\Delta \varphi_{min}-1/\sqrt{N}$) are plotted in Figure 3b. The plot reveals that the mixing between coherent and squeezed state, together with the parity detection, leads to surpassing the SQL. As a result, the sensor architecture of Figure 1 is suitable for operating in the super-sensitivity regime. Moreover, the appropriate choice of the coherent and squeezed parameters can induce the chem/bio sensor to operate at the Heisenberg precision limit (HL). In this sense, Figure 3c shows the locii of the points for which $\Delta \varphi_{min}$ approaches HL. The plot records two branches. The first corresponds to high and low values of r and $\left|\alpha_{0}\right|$, respectively. The second involves also lower values of r, and for this reason can be assumed as a guideline for the design of the MRR source, values that are convenient for such a source to operate with low pump powers.

It is generally recognized that the SFWM sources, if compared with true single photon emitters (trapped ions or quantum dots) present the following drawback: the quantum state produced is a squeezed state where the generation process is probabilistic, and a single photon pair generation can be approximated if the pump laser is relatively weak [30,31]. However, if this can be considered a limitation in several quantum applications, in our scenario the MRR source based on the SFWM can be considered as an efficient choice in order to obtain the design condition given by Figure 3c.

Although SFWM is efficient and useful in silicon and silicon-enriched SiN waveguides, other detrimental nonlinear phenomena can be also excited. In particular, in silicon-based waveguides, both TPA and FCA increase the propagation loss, inducing a reduction of the enhancement factor of the ring resonator. Moreover, the MRR source can be affected by resonance shifts due to the plasma effect, resulting in a reduction of the squeezing parameter. However, if the pump powers are kept low enough to suppress the cross-TPA and FCA effects, we can maximize the total flux of the generated signal photons according to Equation (8) [32,33], where the squeezing parameter is calculated as a function of the MRR field enhancement factor F:

(8) $$r=\sqrt{\frac{\pi }{4}}\gamma \sqrt{P_{1}}\sqrt{P_{2}}L{\left|F\right|}^{4}\Theta \left(\Delta \beta \right)$$

The phase matching effects are included in the function Θ. The terms γ and L designate the Kerr nonlinear parameter and the cavity length, respectively.

We know that the silicon-enriched SiN on SiO_2_ platform does not suffer from the pump power limitation, due to the negligible TPA effect. Thus, for the other two platforms (SOI, SOS) considered here, we can assume Equation (8) as valid by considering negligible the cross-TPA of the generated signal photons [33]:

(9) $$P_{0}\ll P_{TPA}=\frac{{\left(\gamma L\right)}^{-1}}{{\left|F\right|}^{2}\beta_{TPA}}\frac{2\pi n_{2}}{\lambda }$$

where $\beta_{TPA}$ and $n_{2}$ are the TPA coefficient, and the Kerr nonlinear refractive index, respectively. Similarly, for a CW excitation, the FCA effect can be considered negligible if the following condition is satisfied [33]:

(10) $$P_{0}\ll P_{FCA}=\sqrt{\frac{4\left(\hbar \omega_{p1}\cdot \hbar \omega_{p2}\right)A_{eff}^{2}}{\left(\hbar \omega_{p1}+\hbar \omega_{p2}\right)\beta_{TPA}\tau_{c}\sigma_{FCA}L}}\frac{1}{{\left|F\right|}^{2}}$$

where $A_{eff}$, $\sigma_{FCA}$, and $\tau_{c}$ are the effective modal pump area, the FCA cross section, and the effective recombination lifetime.

## 5. Design Rules for SOI, SOS, SiN Platforms

The nonlinear parameters for silicon are given in [34,35], where the wavelength dispersion of the nonlinear parameters is also taken into account. The operative wavelengths are around 1550 nm, 1330 nm, and 640 nm for SOI, SOS, and SiN platforms, respectively. In addition, the waveguide sizes H×W are 220 nm × 500 nm for SOI and SOS platforms, and 340 nm × 900 nm for the SiN platform. The electromagnetic field simulations inside the waveguides have been performed by means of a commercial software based on full-vectorial FEM [36] and the home-made software code. In our procedure the FEM electromagnetic module is used to evaluate the electric field distributions inside the SOI, SOS, and SiN waveguides, in order to determine optical features as propagation constant and group velocity. According to [37], these parameters have been then used as inputs to the resonator equations implemented by means of the home-made software code in order to predict the spectral response and the quality factor Q. Finally, the field enhancement factor F is evaluated as: $F=\kappa_{c}/\left(1-e^{-0.5\alpha L}\sqrt{1-\kappa_{c}^{2}}\right)$, where α is the linear propagation loss coefficient and the coupling factor $\kappa_{c}^{2}$ is the fraction of the input powers coupled in the MRR.

**Figure 4:**
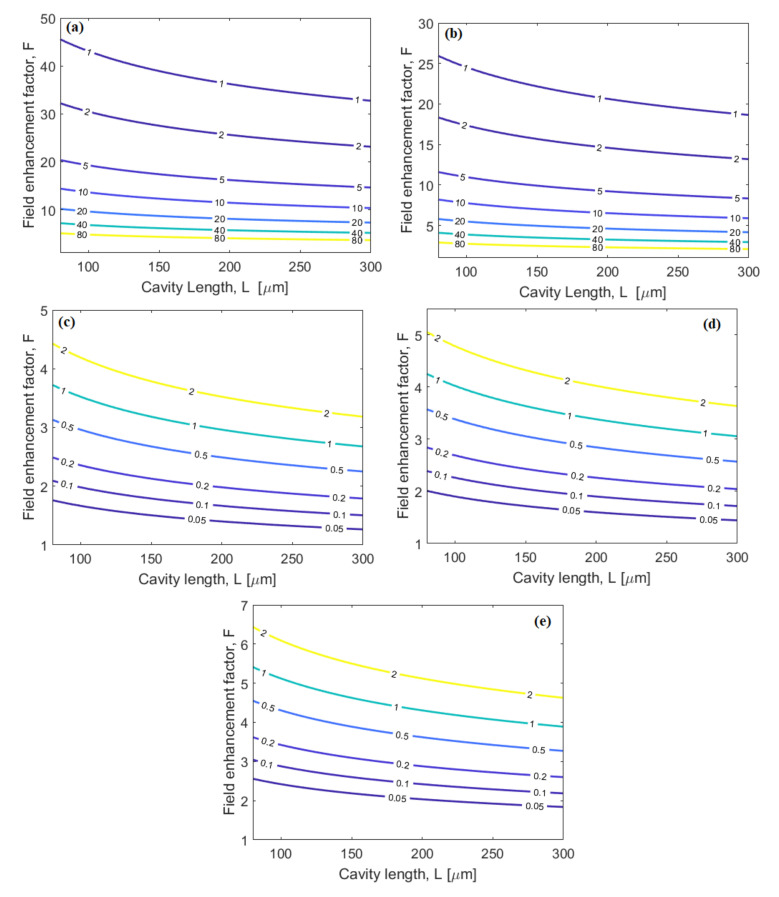
(**a**,**b**) Level curves of $min\left\{P_{TPA},P_{FCA}\right\}$ in the plane (L,F), silicon-on-insulator (SOI) and silicon-on-sapphire (SOS) platforms, respectively. The values are expressed in mW; (**c**,**d**,**e**) level curves of squeezing parameter r, in the plane (L,F), for SOI, SOS, and silicon nitride-on-SiO_2_-on Si (SiN) platforms, respectively.

Figure 4a,c show the level curves for the $min\left\{P_{TPA},P_{FCA}\right\}$ in the plane (L,F), for SOI and SOS, respectively. In the simulations we have assumed the linear propagation loss equal to 0.45 ± 0.12 dB/cm for SOI and SOS waveguides [38] and 1 dB/cm for the SiN platform [27,28,29]. The curves give indications of the MRR features and the pump powers needed to avoid the detrimental effects of TPA and FCA. Indeed, if we suppose operation with $P_{1}=P_{2}$ = 0.4 mW, then the conditions of Equations (9) and (10) are satisfied ($P_{1}=P_{2}<min\left\{P_{TPA},P_{FCA}\right\}\;/10$) if the racetrack ring resonator is designed with F< 20 or 12 for SOI or SOS, respectively, and with a cavity length ranging between 80 μm and 300 μm. It is worth outlining that the condition $P_{1}=P_{2}<min\left\{P_{TPA},P_{FCA}\right\}\;/10$ has been adopted as a design rule in order to operate in the regime of negligible TPA and FCA effects. In this context, Figure 4b,d,e show the level curves for the squeezing parameter (r), for SOI, SOS, and SiN platforms, respectively. The F axis has been zoomed-in in order to evidence r up to 2. Thus, these curves together with Figure 3c lead to designing the MRR source so as to guarantee a sensor sensitivity at the Heisenberg limit. The designed parameters are summarized in Table 1, where we have chosen to operate with $P_{1}=P_{2}$ = 0.4 mW, r = 0.3 and 0.9, inducing an coherent state parameter $\left|\alpha_{0}\right|$=1.3 and 1.982, respectively (see Figure 3c). The above mentioned values leads us to operate with negligible TPA and FCA effects, having a cavity length L = 100 and 180 μm for r = 0.3, and 0.9, respectively. Results in Figure 4, where the level curves are shown as a function of cavity length and field enhancement factor that, in its turn, depends on the resonator-waveguide power coupling coefficient (factor $\kappa_{c}^{2}$), represent a first order tolerance analysis, too. Other possible parameter variations, such as the waveguide cross section tolerance, would represent a second order effect, not significant in terms of minimum power and squeezing parameter requirements needed for this kind of quantum sensors.

The values listed in Table 1 indicate that, under the design condition $\Delta \varphi_{min}=1/N$ (HL achieved), the sensitivity improves while the squeezed parameter is increased. However, in case of a biosensor the detectable concentration of the analyte in the medium should be, typically, down to pg/ml. As a result, the detection limits (the fraction of the effective refractive index units, RIU) should be up to 10^−7^ RIU. These values are already achieved with photonic sensors operating in the classical regime. Surely, the mentioned values could be obtained using a quantum sensor if the quantum resources were strongly increased. However, in this context, the main goal of the quantum approach is compromised to recover the phase value by measurement, using a limited amount of resources.

**Figure 5:**
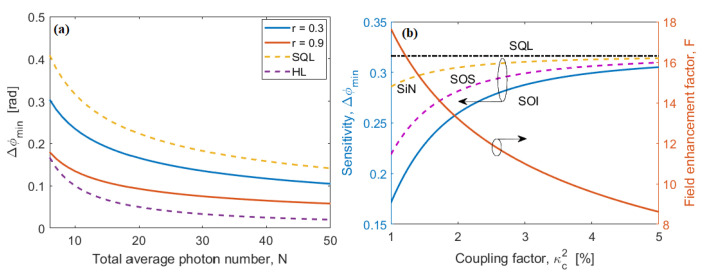
(**a**) $\Delta \varphi_{min}$ as a function of the total average photon number, for r = 0.3, and 0.9, respectively; (**b**) $\Delta \varphi_{min}$ as a function of the coupling factor, $\kappa_{c}^{2}$, for SOI, SOS, and SiN platforms, respectively, assuming N = 10 and L = 100 μm.

The results of Figure 5a (see curve for r = 0.9) indicate that 3.32 × 10^−5^ RIU can be obtained with the quantum approach, assuming the total average photon number, N = 10, λ = 1550 nm and the sensing length of 1 mm. On the basis of the cited RIU values, the quantum approach seems to give worst performance with respect to the photonic sensors operating in classical regime. However, some further comments are noteworthy. For biosamples which start to degrade at 100 nW of optical power (large number of photons), the quantum optical sensing can be non-relevant, since the large numbers of photons used in the classical approach guarantees better sensitivity with respect to the quantum regime operating with a limited amount of quantum resources (the total average photon number, N). However, an alternative comparison can be made if the total average photon number is fixed for both approaches. In this context, the best sensitivity obtained under classical regime is 7.8 × 10^−5^ RIU for the total average photon number N = 10, λ = 1550 nm, and the sensing length of 1 mm. Thus, the classical limit is introduced as a reference level to find the design rules in order to obtain both super-resolution and super-sensitivity, representing the ultimate possible performance in the quantum scenario. Moreover, the field of application of a quantum sensor is not in the regime of 100 nW, but rather where a limited number of photons are needed and where the classical approach is forbidden by realistic tasks. As outlined in Ref. [2], the quantum sensing can manifest its potentiality in applications such as the detection of α-synuclein, which is gaining relevance as a potential biomarker for Parkinson’s disease. Finally, a variety of relevant chem/bio sensing scenarios such as label free rare biomarker detection in fluids may benefit from the sensitivity and resolution promised by optical sensing platform combined with quantum approach. In the context, the aim of the present work is to demonstrate theoretically the possibility to realize an integrated photonic sensor able to reach both super-sensitivity and super-resolution. Moreover, we believe that a further novelty is represented mainly by the use of the parity detection approach integrated on the chip and operating at room temperature. 

The $\Delta \varphi_{min}$ as a function of N for different values of r are plotted in Figure 5a. We record that our sensor works always in the super-sensitivity regime where $\Delta \varphi_{min}$ < SQL. Moreover, the Heisenberg limit is tangentially reached for low values of N, and these values increasing with the squeezing parameters. Figure 5b shows the $\Delta \varphi_{min}$ as a function of the coupling factor $\kappa_{c}^{2}$ (fraction of the input powers coupled in the MRR), assuming N = 10 and L = 100 μm, for SOI, SOS and SiN platforms, respectively. The curves indicate that for N = 10 and for values of $\kappa_{c}^{2}$ commonly used, the Heisenberg limit is not reached, although the SQL is exceeded.

**Figure 6:**
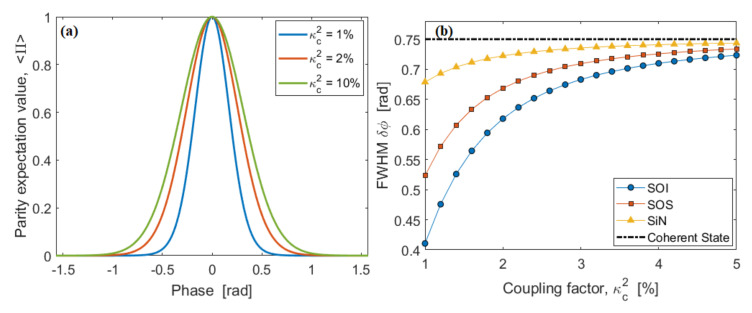
(**a**) Parity expectation value, 〈Π〉, as a function of the phase φ, for different values of the coupling factor, $\kappa_{c}^{2}$, assuming SOI platform, N = 10 L = 100 μm, and $P_{0}$ = 0.4 mW; (**b**) Resolution (δφ) as a function of the coupling factor, $\kappa_{c}^{2}$, for SOI, SOS, and SiN platforms, respectively, assuming N = 10 and L = 100 μm. The dashed-dot black line represents the value for coherent state interferometry.

The expectation value of the parity operator 〈Π〉 as a function of the phase φ for different values of coupling factor $\kappa_{c}^{2}$ is plotted in Figure 6a, assuming the SOI platform, N = 10, $P_{0}$ = 0.4 mW, and L = 100 μm. Our sensor works in the super-resolution regime where the curve width (FWHM:δφ) is always lower than the value obtained with classical interferometry [24]. Moreover, the plot shows that δφ decreases with the coupling factor $\kappa_{c}^{2}$, as a result of the increasing contribution of the squeezed state. Further improvement is obtained by increasing the resource N. Similar trends are recorded from SOS and SiN platforms, but not plotted in Figure 6a for editing reasons. The squeezing state effect is better evidenced in Figure 6b, where the width δφ is shown as a function of the coupling factor $\kappa_{c}^{2}$, assuming N = 10, $P_{0}$ = 0.4 mW, and L = 100 μm, for SOI, SOS, and SiN platforms, respectively. In the figure, the curve width in the case of coherent state interferometry is also plotted for comparison.

At this step some comments on the pump filtering are worth making. Considerable efforts have been made to achieve on-chip high extinction filters for quantum photonics. The proposed solutions typically involve Bragg gratings [39], arrayed-waveguide gratings [40], cascaded Mach-Zehnder interferometers (MZIs) [41], and coupled-resonator optical waveguides [5], [42]. Although it has been proven that all solutions record ~100 dB extinction, the AWG may not be favorable for scalability. While the solution proposed in [39] records both high extinction and compactness, we think it could present non-trivial limitations when applied to the case of two-colors pump, as in our scenario. Thus, we think that, due to the advantages of compact footprint, flat-top passbands, high extinction, and multiple stop-bands [5], the coupled-resonator optical waveguides can be the best solution for the quantum integrated sensor presented here. In particular, for all the three considered platforms, we can assume racetrack resonators (see the inset of Figure 1) having a total length ($L_{f}$) less than that of the SFWM MRR source. Thus, on the basis of the previous investigations, it is acceptable to assume $L_{f}$ around 70 μm. Moreover, the filter performance is strongly dependent upon the coupling coefficients between the ring resonators. In this sense, we propose here to use the maximally flat design, where the coupling coefficients are chosen on the basis of the Butterworth polynomial. Thus, setting $\kappa_{c,i}^{2}$ the coupling factor between the *i-th* and *i+1-th* race-track resonator, we have, in case of five coupled resonators, $\kappa_{c,1}^{2}$= $\kappa_{c,4}^{2}$= 0.0955$\kappa_{c,0}^{4}\times v_{g}/L_{f}$; $\kappa_{c,2}^{2}$=$\kappa_{c,3}^{2}$= 0.0295$\kappa_{c,0}^{4}\times v_{g}/L_{f}$, where $v_{g}$ and $\kappa_{c,0}^{2}$ are the group velocity and the coupling factor between the input bus and the first resonator of the array, respectively. Five coupled resonators can guarantee extinction values ranging between −40dB and −60 dB. Thus, according to the measurements proposed in [5], 100 dB extinction can be obtained by cascading two building block each with 5-coupled resonators.

Finally, considering the thermo optic effects for both Si and SiN materials, we estimate a resonance wavelength shift of 117.8 pm/K and 7.63 pm/K, for SOI and SiN platforms, respectively. According to the results of Figure 5 and Figure 6, where a cavity length of 100 μm is considered, we calculate a HWHM of 138.52 pm and 19.1 pm for SOI and SiN platforms, respectively. In this context, we think that a temperature change of 1.18 K (SOI) and 2.5 K (SiN) is compatible with the SFWM effect. Since the MRR sources are sensitive to thermal effects, we believe that a thermoelectric controller should be needed to maintain the chip temperature and ensure a temperature stability better than 0.01 K.

## 6. Chip-Integrated Parity-Based Detection Technique

Generally speaking, parity can be directly measured by counting the number of photons at one MZI output. This one-output approach requires the use of single-photon-counting detectors (SPDs). For the SOI and SOS platforms, the most efficient option is provided by superconducting nanowire single-photon detectors (SNSPDs), which are sensitive at a wide range of wavelengths, including the 1550 nm band. Although they realize near-ideal detection, a cryogenic temperature is required [30,31], making them unsuitable for full integration on chip. Recently, single photon avalanche detectors (SPADs) near room temperature have been demonstrated at 1330 nm and 1550 nm, using a vertically coupled Ge APD and a waveguide butt-coupled GeSn APD, respectively [43,44]. In the current state-of-the-art, their performances are limited by the dislocation density at the Si interface. However, there is hope that those SPADs could fill the 300K integrated role with sufficient performance. Conversely, the SiN platform does offer good possibilities for full integration of SPADs on the sensor. Indeed, a theoretical investigation of a 16 μm long silicon rib-waveguide SPAD with an absorption of >99% at 640 nm, end-fire coupled from an input silicon nitride rectangular waveguide, recorded dark count performance of <4 kcps at 300K and <5 cps at 243K [45]. However, our goal here is not to use any SPADs, and to instead attain parity detection using standard photodiodes. We investigated several waveguide-circuit parity architectures and we found a method of obtaining parity directly, without recourse to photon-number-resolving detectors. The technique was presented in [23] where a homodyne detection scheme was applied when two mode squeezed vacuum light state arrived at the MZI input. However, here we propose a different protocol as a result of the presence of mixed quantum states. In this sense Equation (6) can be written as:

(11) $$\langle \hat{\Pi }\rangle =\frac{e^{\left[-F\right]}}{\sqrt{\frac{4{\left|\langle c^{†}\rangle \right|}^{2}\left(\langle c^{†}c\rangle -{\left|\langle c^{†}\rangle \right|}^{2}\right)}{\left({\left|\langle c^{†}\rangle \right|}^{2}+{\left|\langle d^{†}\rangle \right|}^{2}\right)}+1}}$$

where

(12) $$F=\frac{\left(2{\left|\langle c^{†}\rangle \right|}^{2}+4{\left|\langle c^{†}\rangle \right|}^{2}\left({\left|\langle c^{†}\rangle \right|}^{2}-\langle c^{†}c\rangle \right)+4\left(\langle c^{†2}\rangle \langle c\rangle +{\langle c^{†}\rangle }^{2}{\langle c\rangle }^{2}\right)\right)}{\frac{4{\left|\langle c^{†}\rangle \right|}^{2}\left(\langle c^{†}c\rangle -{\left|\langle c^{†}\rangle \right|}^{2}\right)}{\left({\left|\langle c^{†}\rangle \right|}^{2}+{\left|\langle d^{†}\rangle \right|}^{2}\right)}+1}$$

**Figure 7:**
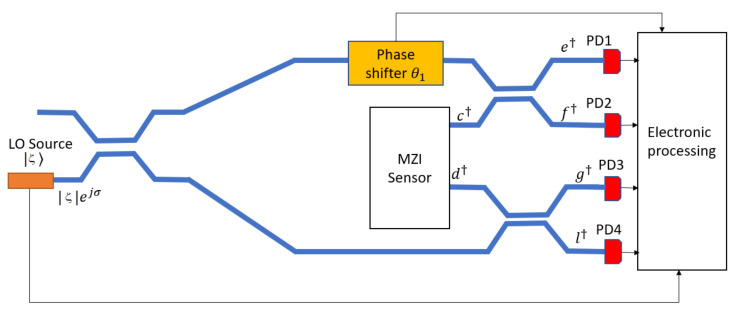
Integrated circuit for parity detection.

Thus, in order to recover Equations (11) and (12) we need to calculate all the expectation values by means of different measurements, following a well-defined protocol. In this context, the parity detection architecture integrated with the chem/bio sensor chip should be as sketched in Figure 7, where the local oscillator (LO, a strong coherent beam of known intensity ${\left|\varsigma \right|}^{2}$ and phase σ) is used in order to perform independent measurements. Four waveguide-integrated photodetectors (that are well known or “standard” in the art of the specific platform) are then placed at the output ports of this circuit. The protocol, proposed here, involves measurements of $Y_{1}\left(\left|\varsigma \right|,\sigma \right)$, $Y_{2}\left(\left|\varsigma \right|,\sigma \right)$, $X_{1}\left(\left|\varsigma \right|,\sigma \right)$ and $X_{2}\left(\left|\varsigma \right|,\sigma \right)$ of $\langle e^{†}e-f^{†}f\rangle$, $\langle g^{†}g-l^{†}l\rangle$, $\langle e^{†}e+f^{†}f\rangle$, and $4\langle e^{†}ef^{†}f\rangle$, respectively. Thus, performing two measurements of $Y_{1}\left(\left|\varsigma \right|,\sigma \right)$ and $Y_{2}\left(\left|\varsigma \right|,\sigma \right)$ (intensity difference at the photodetectors PD1, PD2 and PD3, PD4) (i.e., $Y_{1}\left(\left|\varsigma \right|,0\right)$, $Y_{1}\left(\left|\varsigma \right|,\pi /2\right)$, and $Y_{2}\left(\left|\varsigma \right|,0\right)$, $Y_{2}\left(\left|\varsigma \right|,\pi /2\right)$), we obtain the expectation values of $\langle c^{†}\rangle$, 〈c〉, and $\langle d^{†}\rangle$, 〈d〉, respectively. Similarly, the intensity summation at the detectors PD1, PD2 (i.e., $X_{1}\left(\left|\varsigma \right|,\sigma \right)$ measurement) leads us to extract the term $\langle c^{†}c\rangle$. Finally, four measurements on $4\langle e^{†}ef^{†}f\rangle$ (or are performed when setting the phase of $\theta_{1}$=0, π/4,- π/4, and π/2 in order to calculate $\langle c^{†2}\rangle$. The homodyne technique has been also recently proposed [46]. Finally, we propose that the phase shifter ($\theta_{1},\;\theta_{2}$) can be realized by means of the p-i-n structure for SOI and SOS platforms [47] and by using thin film PZT on SiN in the case of the SiN platform [48]. Considering an overview of parity detection, the on-chip integrated parity detection scheme presented in Figure 7 has two important aspects: (1) the four integrated photodetectors are conventional (not single-photon) photodiodes; (2) our approach more generally applies to other quantum photonic chips in both the quantum metrology and quantum communications areas. However, some further comments are noteworthy. The cryogenic lab equipment has become more and more affordable and reliable, especially in the last five years. However, we guess that operation at room temperature is still an open challenge, for a number of applications. In this context, the limiting devices are the photodetectors that must operate at cryogenic temperature in order to meet the requirements on the dark count rate. This is particularly true in the case of detection schemes based on coincidence measurements. Our beginning statement is preparatory to introduce the novelty of the parity detection scheme on the integrated platform, avoiding the photon number counting approach and then operating with standard photodetector at room temperature. However, despite the non-trivial advantage to operate at room temperature, the parity detection scheme suffers from two main drawbacks if compared with the coincidence measurements at cryogenic temperature: (i) a more architecture complexity, (ii) larger processing time. On the other hand, we think that the loss problem is very critical for quantum sensing based on coincidence measurement scheme (i.e., based on N00N state). As detailed in Ref. [23], the potential advantage of the parity detection is the metrology in presence of loss. In this sense, we think that our approach, using parity detection and mixing between squeezed and coherent states, could operate under loss condition. However, we are aware that our work can be considered as a first step in the direction to realize quantum integrated sensor operating at room temperature. In this sense, we believe that a future merging between theory and experimental setup could give a further contribution on this research topic. Finally, the architectures of Figure 1 and Figure 7 are suitable variations in which the beam splitters and the beam combiners are substituted by MMI devices [49].

## 7. Conclusions

For operation at 1550 nm, 1330 nm, and 640 nm, chip-scale room-temperature integrated quantum chem/bio sensors have been proposed for foundry implementation in the Group IV technological platform. In particular, SOI, SOS, and SiN platforms have been investigated. The focus of this work has been to determine the design rules allowing operation in the super-sensitivity regime with respect to the classical approach. Basically, the devices proposed present the one-chip co-integration of a coherent source input, a squeezed vacuum source (i.e. racetrack microring resonator in which signal photons are generated by means of the SFWM process), the MZI sensor, and an integrated photonics circuit to implement the homodyne technique in order to perform parity measurements. The proposed device achieves the super-sensitivity condition due to the mixing between single-mode squeezing (induced by the degenerate SFWM) and coherent states.

## Figures and Tables

**Table 1:** Sensor designed parameters.

Parameters	SOI	SOS	SiN
Cavity length, L = 100 μm; r = 0.3			
$n_{sq}$	0.0927	0.0927	0.0927
$n_{co}$	1.69	1.69	1.69
Sensitivity, $\Delta \varphi_{min}$	HL = 0.57	HL = 0.57	HL = 0.57
Field enhancement factor, F	2.6	2.98	3.8
Cavity quality factor, Q	5595	8517.6	16751
Signal wavelength, $\lambda_{s}$	1550 nm	1330 nm	640 nm
Pump-1 wavelength, $\lambda_{p1}$	1544.1 nm	1325.7 nm	638.271 nm
Pump-2 wavelength, $\lambda_{p2}$	1555.9 nm	1334.3	641.7380 nm
Cavity length, L = 180 μm, r = 0.9			
$n_{sq}$	1.0537	1.0537	1.0537
$n_{co}$	3.9283	3.9283	3.9283
Sensitivity, $\Delta \varphi_{min}$	HL = 0.2	HL = 0.2	HL = 0.2
Field enhancement factor, F	2.97	3.38	4.305
Cavity quality factor,Q	13123	19861	38621
Signal wavelength, $\lambda_{s}$	1550 nm	1330 nm	640 nm
Pump-1 wavelength, $\lambda_{p1}$	1546.7 nm	1327.6 nm	639.0388 nm
Pump-2 wavelength, $\lambda_{p2}$	1553.3 nm	1332.4 nm	640.9640
